# Selection of reference genes in different myocardial regions of an *in vivo *ischemia/reperfusion rat model for normalization of antioxidant gene expression

**DOI:** 10.1186/1756-0500-5-124

**Published:** 2012-02-29

**Authors:** Nicoletta Vesentini, Cristina Barsanti, Alessandro Martino, Claudia Kusmic, Andrea Ripoli, AnnaMaria Rossi, Antonio L'Abbate

**Affiliations:** 1 Istituto di Fisiologia Clinica, Consiglio Nazionale delle Ricerche, Pisa, Italy; 2 Scuola Superiore Sant'Anna, Pisa, Italy; 3 Dipartimento di Biologia-Sezione Genetica, Università di Pisa, Pisa, Italy; 4 Fondazione Toscana "Gabriele Monasterio", Pisa Italy

## Abstract

**Background:**

Changes in cardiac gene expression due to myocardial injury are usually assessed in whole heart tissue. However, as the heart is a heterogeneous system, spatial and temporal heterogeneity is expected in gene expression.

**Results:**

In an ischemia/reperfusion (I/R) rat model we evaluated gene expression of mitochondrial and cytoplasmatic superoxide dismutase (*MnSod, Cu-ZnSod*) and thioredoxin reductase (*trxr1*) upon short (4 h) and long (72 h) reperfusion times in the right ventricle (RV), and in the ischemic/reperfused (IRR) and the remote region (RR) of the left ventricle. Gene expression was assessed by Real-time reverse-transcription quantitative PCR (RT-qPCR). In order to select most stable reference genes suitable for normalization purposes, in each myocardial region we tested nine putative reference genes by geNorm analysis. The genes investigated were: Actin beta (*actb)*, Glyceraldehyde-3-P-dehydrogenase *(gapdh)*, Ribosomal protein L13A *(rpl13a)*, Tyrosine 3-monooxygenase *(ywhaz)*, Beta-glucuronidase *(gusb)*, Hypoxanthine guanine Phosphoribosyltransferase 1 *(hprt)*, TATA binding box protein *(tbp)*, Hydroxymethylbilane synthase *(hmbs)*, Polyadenylate-binding protein 1 *(papbn1*). According to our findings, most stable reference genes in the RV and RR were *hmbs/hprt *and *hmbs/tbp/hprt *respectively. In the IRR, six reference genes were recommended for normalization purposes; however, in view of experimental feasibility limitations, target gene expression could be normalized against the three most stable reference genes (*ywhaz/pabp/hmbs*) without loss of sensitivity. In all cases *MnSod *and *Cu-ZnSod *expression decreased upon long reperfusion, the former in all myocardial regions and the latter in IRR alone. *trxr1 *expression did not vary.

**Conclusions:**

This study provides a validation of reference genes in the RV and in the anterior and posterior wall of the LV of cardiac ischemia/reperfusion model and shows that gene expression should be assessed separately in each region.

## Background

Cardiac muscle is a heterogeneous system and many parameters such as blood flow and perfusion [1-3], patterns of ion channel activation [4-6] differ in distinct heart regions. As gene expression is concerned, spatial heterogeneity between cardiac chambers as well as between left and right ventricle have long been recognized [7,8]. However, mounting evidences suggest that also conduction velocity, repolarization heterogeneities, and arrhythmia susceptibility in different left ventricle (LV) regions can be attributable to regional differences in their protein expression pattern and function [9,10]. The spatial, functional and temporal heterogeneity that is distinctive becomes especially relevant in the injured heart [11-13].

*In vivo *occlusion of the left anterior descending (LAD) coronary artery followed by reperfusion is extensively used as an animal model of ischemic heart disease. Upon coronary obstruction, restoration of blood flow to the ischemic myocardium modulates the size of myocardial infarct and the chance of cell survival. However, this process, termed reperfusion, *per se *can also induce injury. The exact mechanism of reperfusion injury has not yet been clarified, although it probably involves cellular overload of calcium, mitochondrial impairment and oxidative stress-induced damage [14]. The role of endogenous antioxidants in reperfusion injury has been studied extensively, although results are not always consistent (for a review see [15,16]). In fact, activity or gene expression of antioxidant enzymes has been reported to either increase or decrease upon ischemia/reperfusion (I/R) [17-23]. This may be due to different experimental conditions and/or to variation of cardiac endogenous antioxidant expression at different times of reperfusion [20].

Although experimental *in vivo *ischemia most commonly involves mono-vasal occlusion, very few investigations have been addressed to comparative analysis on tissues from different LV regions [9,11-13], as most reports on small animal models analyzed the total or partial left ventricular tissue [24-26] or even both ventricles combined [27].

The working hypothesis of the present study is that gene expression analysis performed separately in LAD territory and in the remaining cardiac regions is required as a prior condition for an accurate study of the effects of ischemia and reperfusion.

Real-time reverse-transcription quantitative PCR (RT-qPCR) is the method of choice for analyzing gene expression [28]. However, selection of appropriate internal reference genes or housekeeping genes is necessary for reliable results in RT-qPCR. Reference gene expression should remain constant in the tissues of interest [29] and in the established experimental conditions. The lack of these requirements may lead to erroneous or inaccurate results [30-33].

Previously, single reference genes have been widely used to normalize expression of the target genes. However, numerous reports have stated that classic reference genes may vary extensively in different experimental conditions and tissues and are therefore unsuitable for normalization purposes in the absence of an accurate validation [32,34,35]. For example, one of the most traditionally used genes for normalization has been *gapdh *although several publications show that its expression is variable and not suitable for normalizing mRNA levels [36-38].

Normalization against multiple internal reference genes has now become a prerequisite for correct expression analysis [39] and software programs devoted to evaluation of expression stability and selection of the most suitable reference genes under different experimental conditions have been developed [40,41]. This requirement is paramount in a complex tissue such as the myocardium that is composed by multiple cell types and especially during ischemia-reperfusion where also not specific RNA degradation can take place.

In an *in vivo *rat model of myocardial I/R we focused on gene expression of three antioxidant enzymes ubiquitously expressed--mitochondrial and cytosolic superoxide dismutase (*MnSod *and *Cu-ZnSod *respectively) and cytosolic thioredoxin reductase (*trxr1*)--whose role in the protection of ischemia/reperfusion injury has been investigated extensively [16,42,43]. Short (4 h) and long (72 h) reperfusion times were considered in order to evaluate the role of these antioxidant enzymes during two different phases of cardiac wound healing: the necrosis/apoptosis and the proliferation phase respectively [44,45].

The first endpoint of our study was to evaluate a set of candidate reference genes for their use in normalizing RT-qPCR data in three distinct regions of the heart, namely the right ventricle, the central LAD ischemic/reperfused area of the left ventricle, and its undamaged posterior wall.

The second endpoint of the study was to verify alterations in *MnSod, Cu-ZnSod *and *trxr1 *gene expression level upon ischemia/reperfusion-induced oxidative stress in the different heart areas at the two different times of reperfusion.

## Results and discussion

### Selection of reference genes

**Figure 1:**
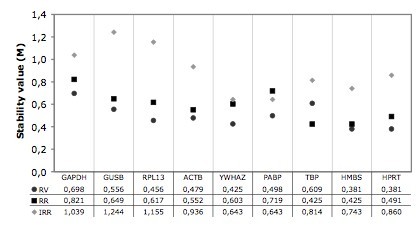
**Average expression stability values of the candidate reference genes in the different myocardial regions**. Average expression stability values (M) of nine candidate reference genes as calculated by geNorm software. RV, Right Ventricle; RR, Remote Region of the left ventricle; IRR, Ischemic/Reperfused Region of the left ventricle.

geNorm software was used to test the candidate reference genes in order to rank them on the basis of their expression stability value (M). The M value is the average pairwise variation of a particular gene with all other reference genes [40]. The lowest M value corresponds to the most stable reference gene, while the highest corresponds to the least stable. geNorm analysis of expression stability showed differences in gene expression in the three myocardial regions (Figure 1). In all cases, stability gene values were always below the 1.5 cut-off set by the algorithm, thereby signifying stable expression levels for all genes. In particular, analysis showed that in the RV most stable genes were *hmbs/hprt *(M = 0.38). In the RR of the left ventricle the highest stability was achieved by *hmbs/tbp *(M = 0.42) while IRR had the highest M values, and the most stable genes were *ywhaz/pabp *(M = 0.64).

It is noteworthy that, as previously reported by Brattelid et al., [37] in a rat model of post-infarction heart failure, the highest stability was observed in genes encoding proteins involved in DNA synthesis/transcription, independently of the myocardial area analyzed, thus confirming that they are a suitable alternative to the widely used metabolic gene *gapdh *as reference genes

**Figure 2:**
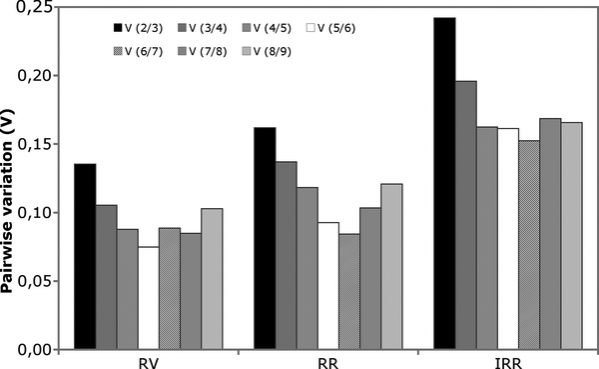
**Pairwise variation of candidate reference genes**. Pairwise variation (V_n_/_n+1_) was analyzed between the normalization factors NF(n) and NF(n + 1) by geNorm software to determine the optimal number of reference genes required for RT-qPCR data normalization the Right Ventricle (RV) (n = 20), Left Remote Region (RR) (n = 20) and Ischemic/Reperfused Region (IRR) (n = 20). In the RV, V_2/3 _is 0.135, and the two genes hmbs and hprt are sufficient for normalizing gene expression data. In the RR, analysis of pairwise variation shows that three reference genes should be included for gene expression studies in order to obtain a value below 0.15 (V_3/4 _= 0.137). Reference genes in the RR therefore should be *hmbs, tbp, hprt*. Finally, in the IRR, analysis of pairwise variation shows that six reference genes should be included for gene expression studies in order to obtain a value below 0.15 (V_6/7 _= 0.148). Reference genes in the IRR should therefore be *ywhaz, pabp, hmbs, tbp, hprt*, and *actb*.

The optimal number of reference genes recommended as normalization factor in the distinct cardiac regions was calculated with pairwise variation and is shown in Figure 2. Vandesompele et al. [40] set 0.15 as a cutoff value below which inclusion of additional genes is not required. According to this analysis, two genes were sufficient for adequate normalization in the RV (*hmbs *and *hprt*) and three in the RR (decreasing rank of stability: *hmbs, tbp, hprt*). In the IRR the number of reference genes to be included was higher, as expected for the area most affected by biochemical and cellular changes, and the use of six reference genes was recommended for normalization purposes (decreasing rank of stability: *ywhaz, pabp, hmbs, tbp, hprt, actb*). However, as highlighted by Vandesompele and colleagues, 0.15 is an arbitrary value and the number of genes used for geometric averaging is a trade-off between accuracy and practical considerations such as cost limitations and limited amount of sample. Therefore, in order to increase experimental feasibility of regional gene expression analysis, target genes were normalized not only with the six reference genes computed by geNorm, but also with a reduced number of genes obtained by the progressive exclusion of the least stable, down to the three best reference genes.

### Target gene expression analysis

**Figure 3:**
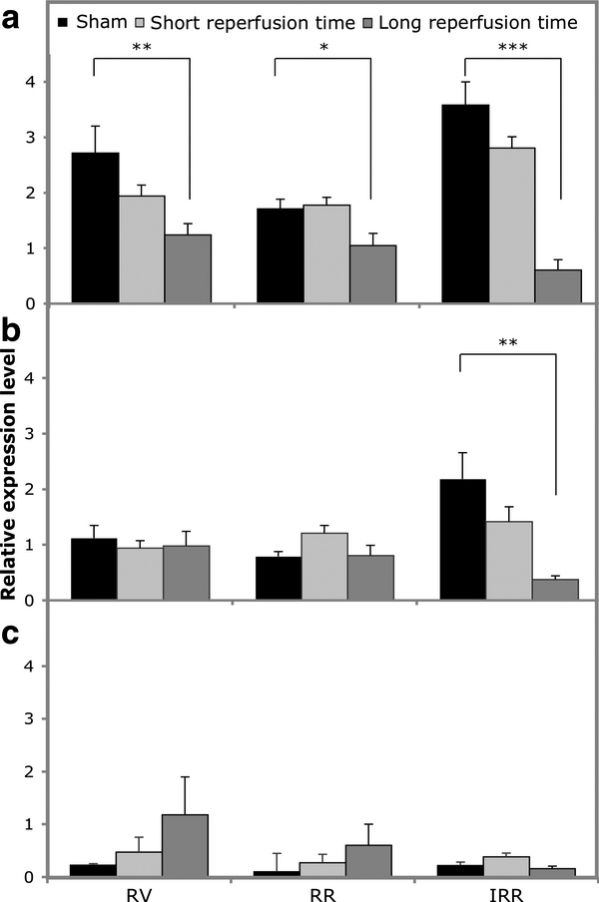
***MnSod, Cu-ZnSod *and *trxr1 *expression levels at different reperfusion times**. *MnSod *(A), *Cu-ZnSod *(B) and *trxr1 *(C) expression levels in short and long reperfusion times upon 30 min of ischemia. Sham animals were pooled together as there were no evident differences between short and long reperfusion times (sham n = 5; short n = 9; long n = 6). Results are expressed as mean ± SE. *p < 0.05; ** p < 0.01; ***p < 0.001.

Expression of *MnSod, Cu-ZnSod *and *trxr1 *was evaluated in the three different cardiac regions in sham-operated and in the short and long reperfused animals, according to the reference genes as indicated by geNorm (Figure 3). As far as the sham group is concerned, we found a significant heterogeneity in the expression level within the two LV areas for both mitochondrial and cytosolic SOD, with the higher levels expressed in IRR (p < 0.05 for both). *trxr1 *expression did not vary among the three ventricular regions.

Regarding *MnSod*, there was an evident drop in expression level in all three cardiac regions upon long reperfusion time only (Figure 3A). In the RV and in the RR *MnSod *expression decreased of 54 and 40% with respect to sham (p < 0.01 and p < 0.05 respectively). In the IRR, expression level decreased of 83% with respect to sham (p < 0.001).

A decrease in *MnSod *activity upon ischemia and reperfusion has been previously described [46,47]. However, our experimental setting disclosed that although a decrease of expression occurs in all cardiac regions, it is greater in the IRR and occurs only in the long reperfusion time, corresponding to the proliferative phase of wound healing during which fibroblasts and endothelial cells proliferate and matrix proteins are produced [44]. On the contrary, *Cu-ZnSod *expression did not vary with respect to sham in the RV and RR at all times. However, in the IRR, after long reperfusion there was a drop of 83% (p < 0.01 vs sham) (Figure 3B).

Finally *trxr1 *expression levels did not vary significantly upon I/R during either short nor long reperfusion with respect to sham in all cardiac regions (Figure 3C).

**Figure 4:**
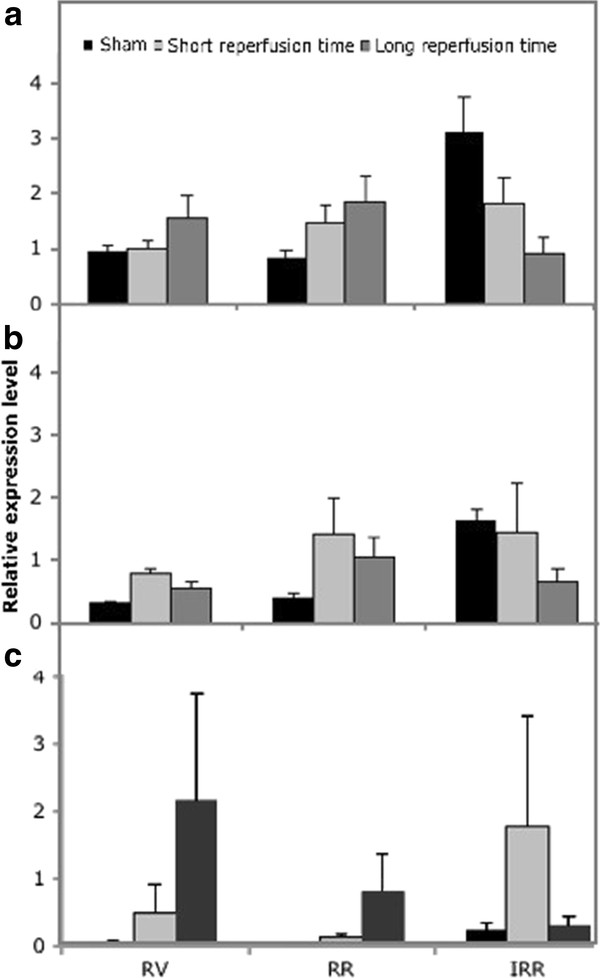
***MnSod, Cu-ZnSod *and *trxr1 *expression levels normalized according to *gapdh***. *MnSod *(A), *Cu-ZnSod *(B) and *trxr1 *(C) expression levels normalized to *gapdh *in sham operated animals and in short and long reperfusion times upon 30 min of ischemia (sham n = 5; short n = 9; long n = 6). Results are expressed as mean ± SE.

To explore the influence of the normalization strategy used we compared the expression of the two target genes that are modulated by I/R normalized either to the subset of genes selected according to geNorm analysis (Figure 3), or to *gapdh *(Figure 4), one of the most frequently used reference gene in the literature. Figure 4 shows that normalization with *gapdh *modified the pattern of expression thereby altering the results observed in Figure 3.

We tested whether results obtained by normalization in the IRR against the six reference genes retained significance also when normalizing with a progressively reduced number of genes. Analysis was performed only on mitochondrial and cytoplasmic SOD, the genes that exhibited a significant variation of expression.

**Figure 5:**
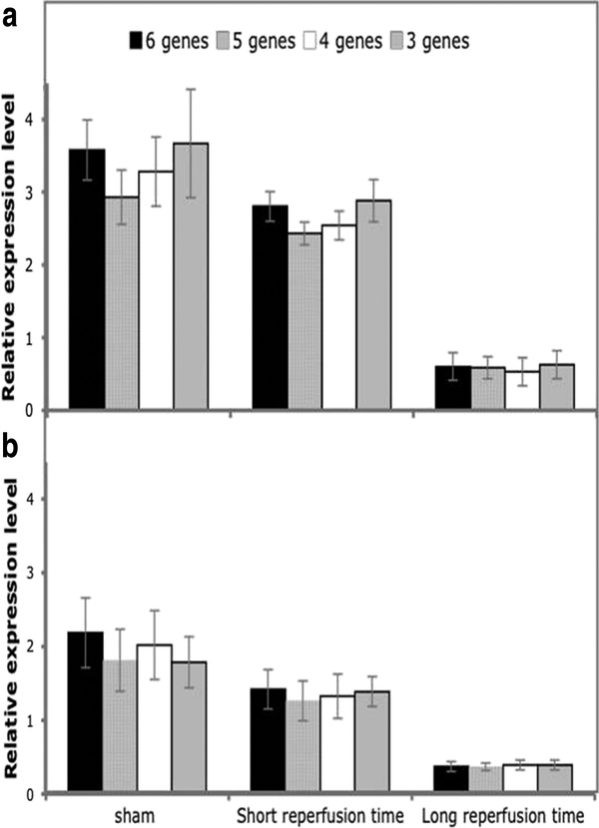
**Normalization of *MnSod *and *Cu-ZnSod *in the IRR with decreasing number of reference genes**. Relative expression of *MnSod *(A) and *Cu-ZnSod *(B) expression level in the IRR with 6, 5, 4 and 3 of the most stable reference genes as indicated by geNorm analysis. (sham n = 5; short n = 9; long n = 6).

Figure 5 shows expression levels of *MnSod *(panel A) and *Cu-ZnSod *(panel B) normalized with 6, 5, 4 and 3 genes. Results did not change when normalization was performed with a reduced number of genes, and when observed, the degree of statistical significance remained the same.

These data suggest that in the rat model of *in vivo *cardiac I/R, expression analysis may be accurately performed by selecting the appropriate reference genes for each region and even reducing the number of reference genes suggested by geNorm analysis. This becomes reasonable considering the hands-on implications (laboratory costs and time) and in consideration of the limiting quantity of the sample that occurs when a spatial analysis is carried out on small-sized experimental models (rats and mice).

## Conclusions

In summary, gene expression of both reference and target genes reflects cardiac heterogeneity in the ischemic and reperfused heart.

geNorm analysis has shown that reference gene stability varies among the three myocardial regions analyzed: *hmbs, hprt *and *hmbs, tbp, hprt *are suitable reference genes in the right ventricle and in the Remote region respectively. Although in the ischemic reperfused region instability is higher, three reference genes could be sufficient for adequate normalization (*ywhaz, pabp, hmbs*).

We show that *Cu-ZnSod *and *MnSod*, but not *trxr1 *expression, varies in the different heart regions during the proliferative phase of post-ischemic wound healing.

Previous investigations report differences in gene expression of antioxidant enzymes in post-infarcted myocardium of rats [13,23,47,48]. However, excluding a few cases [13,48], gene expression is most commonly studied in the whole heart in spite of specific spatial differences in gene expression of both reference and target genes. Whenever a region-specific variability in gene expression occurs, as is the case of *Cu-ZnSod *reported in our study, analysis of the heart as a whole could lead to misleading results by either an over- or under-estimation bias.

A more general survey of spatial and temporal expression of antioxidant-coding genes could offer useful knowledge of the relation between the different phases of cardiac repair as well as constitute possible therapeutic targets.

Although our study was limited to the assessment of antioxidant gene changes related to ischemia-reperfusion, it has a more general value addressing the challenging problems of choice and validation of reference genes which apply to other target genes as well, involved in cardiac pathological processes.

## Methods

### Ischemia/reperfusion model

All experiments were performed according to the guidelines of D.Lgs 116 (1992) and conformed to the "Guiding Principles for Research Involving Animals and Human Beings, " approved by the American Physiological Society.

Twenty male Wistar rats (8-10 weeks, 250-300 g) were anesthetized by intraperitoneal injection of Zoletil 100^® ^+ xylazine (50 mg/Kg and 3 mg/Kg respectively). The heart was exposed through a left lateral thoracotomy and LAD coronary was occluded for 30 min in 15 animals. Then the knot around the vessel was opened and unrestrained reperfusion allowed. At the end of reperfusion, animals were killed. Under deep anesthesia, hearts were arrested in diastole by lethal KCl injection. The hearts were then excised and washed for 10 min with cold Krebs-Henseleit bicarbonate buffer in Langendorff configuration.

Reperfused animals were divided into two groups: "short reperfusion time", which were reperfused for 4 h after the reopening of the LAD (n = 9) and "long reperfusion time", which were reperfused for 72 h after the reopening of the LAD (n = 6).

A control group of sham-operated animals underwent all surgical procedures except for the occlusion of the LAD and were killed in correspondence with the short (n = 3) and the long (n = 2) reperfusion times.

### Tissue harvesting

Hearts were cut below the plane of LAD occlusion and tissue samples were obtained from a) the right ventricle wall (RV), b) the core of the LAD territory, i.e., the ischemic reperfused region (IRR) in the left ventricular wall, c) the left ventricular free wall remote to LAD region (RR). In sham-operated animals tissues were harvested from analogously termed corresponding regions; IRR of sham-operated animals corresponded to the area beside the LAD in the left ventricle. Samples were snap frozen in liquid nitrogen and stored at -80°C until RNA purification was undertaken.

### RNA extraction, quantification and retrotranscription

Frozen samples were transferred to Tri Reagent (Sigma) and homogenized using TissueLyser (Qiagen) according to manufacturer's instructions.

Concentration of RNA was determined by measuring optical density at 260 nm. Integrity of total RNA was assessed by electrophoresis on 1.2% agarose gels. cDNA was obtained from 1 μg of total RNA using the iScript (Bio-Rad Laboratories, Hercules, CA, USA) retrotranscription kit.

### Reference gene selection and real-time PCR

Nine candidate reference genes were selected from those most commonly used in literature and belonging to different functional classes in order to avoid co-regulation. Primers were synthesized by BioFab Research (Roma, Italy). Primer characteristics are described in Table 1.

**Table 1:** Primer sequences of target genes and candidate reference genes for normalization

Gene symbol	Gene name	Accession number	Reference	Forward primer (5'-3')	Reverse primer (5'-3')	Amplic on length	PCR efficiency(%)	Tm (°C)
*actb*	Actin, beta	V01217	[49]	AAGTCCCTCACCCTCCCAAAAG	AAGCAATGCTGTCACCTTCCC	97	106	82.9°C

*ywhaz*	tyrosine 3-monooxygenase/tryptophan 5- monooxygenase activation protein zeta polypetide	NM_013011.2	[49]	GATGAAGCCATTGCTGAACTTG	GTCTCCTTGGGTATCCGATGTC	117	94	77.6°C

*rpl13a*	Ribosomal protein L13A	NM_173340	[49]	GGATCCCTCCACCCTATGACA	CTGGTACTTCCACCCGACCTC	132	104	83.5°C

*gapdh*	glyceraldehyde-3-phosphate dehydrogenase	NM_01708		CTACCCACGGCAAGTTCAAC	CCAGTAGACTCCACGACATAC	138	102	57°C

*gusb*	Glucuronidase, beta	NM_017015		TCACCATCGCCATCAACAACAC	GCTTATGTCCTGGACGAAGTAACC	92	94, 9	59°C

*hprt*	Hypoxantine guanine phosphoribosyl transferase	NM_012583		CCCAGCGTCGTGATTAGTGATG	TTCAGTCCTGTCCATAATCAGTCC	110	104	59°C

*tbp*	TATA box binding protein	NM_001004198		CACCGTGAATCTTGGCTGTAAAC	CGCAGTTGTTCGTGGCTCTC	124	104	58°C

*hmbs*	Hydroxymethylbilane synthase	NM_013168	[50]	TCTAGATGGCTCAGATAGCATGCA	TGGACCATCTTCTTGCTGAACA	76	95, 8	60°C

*Pabpn1*	poly(A) binding protein, nuclear 1	116697	http://medgen.ugent.be/rtprimerdb	TATGGTGCGACAGCAGAAGA	TATGCAAACCCTTTGGGATG	110	95	60°C

*MnSod*	Manganese Superoxide dismutase	NM_017051.2		ATCTGAACGTCACCGAGGAG	TAGGGCTCAGGTTTGTCCAG	141	96	59°C

*Cu.ZnSod*	Copper-Zinc Superoxide dismutase	NM_017050	http://medgen.ugent.be/rtprimerdb	CGAGCATGGGTTCCATGTC	CTGGACCGCCATGTTTCTTAG	101	96	50°C

*txnr1*	Thioredoxin reductase 1	NM_031614.2		GGTGAACACATGGAAGAGCA	GGACTTAGCGGTCACCTTGA	111	98	60°C

Reference and antioxidant-coding gene primer sequences, original references, amplicon sizes, amplification efficiency values and accession number for the PCR analyses in the present study

Real-time PCR was performed using iQ SYBRGreen Supermix (Bio-Rad Laboratories). Reactions contained 1X SYBR Green SuperMix (BioRad), 300 nM of each primer and 100 ng of template in a 25 μl final volume reaction. After an initial denaturation step at 95°C for 3 min, amplification was performed with 40 cycles of denaturation at 95°C for 15 s and annealing at 60°C for 30 s. Amplification was followed by melting curve analysis: a single homogeneous peak confirmed specific amplification for each primer pair.

Relative expression levels of reference genes were determined with the comparative threshold cycle (Cq) method. Relative expression levels of target genes were normalized to the geometric mean of most stable genes as indicated by geNorm software. All samples were run in duplicate and the mean value of each duplicate was used for all further calculations.

Serial cDNA dilution curves were produced to calculate the amplification efficiency for all genes. A graph of threshold cycle vs log_10 _picograms of diluted sample series was produced. The slope of the curve was used to determine the amplification efficiency according to Pfaffl [51]: Efficiency = 10^(-1/slope)^. Amplification efficiency values are reported in Table 1.

Gene expression stability and selection of the most suitable reference genes were evaluated with geNorm analysis. To determine the number of optimal genes required for normalization the software calculated pairwise variation (V_n/n+1_) between Normalization Factor NF_n _and NF_n+1 _[40].

### Statistical analysis

Data are expressed as mean ± SE. Comparisons were made by two-way repeated measures ANOVA. When a significant effect of a factor was indicated, the post-hoc Bonferroni test was used to isolate the statistical differences. Analyses were performed using SPSS 13 (SPSS Inc. Chicago, Il, USA), and a p-value of less than 0.05 was considered statistically significant.

## Competing interests

The authors declare that they have no competing interests.

## Authors' contributions

NV carried out the experiments, analysed the results and drafted the manuscript. CB analysed the results and helped to draft the manuscript. AM participated in the set up of the experiments. CK performed all in vivo experiments and helped to draft the manuscript. AR performed statistical analyses of the data. AMR participated in the design of the study and in its coordination. AL conceived and designed the study. All authors read and approved the final manuscript.
